# Osteosarcoma in Children: Not Only Chemotherapy

**DOI:** 10.3390/ph14090923

**Published:** 2021-09-13

**Authors:** Maura Argenziano, Chiara Tortora, Elvira Pota, Alessandra Di Paola, Martina Di Martino, Caterina Di Leva, Daniela Di Pinto, Francesca Rossi

**Affiliations:** 1 Department of Woman, Child and General and Specialist Surgery, University of Campania “Luigi Vanvitelli”, 80138 Naples, Italy; maurargenziano@gmail.com (M.A.); chiara.tortora@unicampania.it (C.T.); elvira.pota@unicampania.it (E.P.); martina.dimartino@unicampania.it (M.D.M.); caterinadl.94@gmail.com (C.D.L.); daniela.dipinto@unicampania.it (D.D.P.); 2 Department of Experimental Medicine, University of Campania “Luigi Vanvitelli”, 80138 Naples, Italy; alessandra.dipaola92@gmail.com

**Keywords:** osteosarcoma, chemoresistance, therapy, proteasome inhibitors, immunotherapy, iron chelation, lncRNA, TKIs

## Abstract

Osteosarcoma (OS) is the most severe bone malignant tumor, responsible for altered osteoid deposition and with a high rate of metastasis. It is characterized by heterogeneity, chemoresistance and its interaction with bone microenvironment. The 5-year survival rate is about 67% for patients with localized OS, while it remains at 20% in case of metastases. The standard therapy for OS patients is represented by neoadjuvant chemotherapy, surgical resection, and adjuvant chemotherapy. The most used chemotherapy regimen for children is the combination of high-dose methotrexate, doxorubicin, and cisplatin. Considered that the necessary administration of high-dose chemotherapy is responsible for a lot of acute and chronic side effects, the identification of novel therapeutic strategies to ameliorate OS outcome and the patients’ life expectancy is necessary. In this review we provide an overview on new possible innovative therapeutic strategies in OS.

## 1. Introduction

Osteosarcoma (OS) is the most common and severe bone malignant tumor, characterized by altered osteoid deposition and high rate of metastasis [1,2,3,4], which affects especially lungs [5]. It shows a bimodal distribution with the major peak in children and adolescents, as primary bone cancer, and another smaller peak in adults, generally as secondary cancer [5,6]. OS can affect any bone, but it is more frequent at the metaphysis of long bones, where the turnover is very high [7], thus suggesting a correlation between OS onset and rapid bone proliferation, even though its etiology remains not clear. Currently the 5-year survival rate for patients with localized OS is of about 67% [8,9,10,11], while in case of metastases it is inversely proportional to the degree of metastatic state and still remains at 20% [11,12,13].

OS is closely related to its microenvironment, which is involved in tumor growth and dissemination [6,14,15]. It is constituted by immune, bone, vascular and stromal cells, several released molecules, and blood vessels [6,16]. The immune component is mainly represented by tumor-associated macrophages (TAMs) [17], in particular the M2 alternative activated phenotype, and also by T lymphocytes, myeloid cells and dendritic cells [18]. The prevalence of M2 macrophage phenotype in the tumor microenvironment (TME) is generally associated with a poorer 5-year event free survival in patients. However, in literature there are discordant results: the M2 profile should be associated with a reduction in metastasis and an improvement in high-grade OS survival [19]. In 2017, Gomez-Brouchet et al. analyzed the expression of several macrophage biomarkers, observing that CD163-positive M2-polarized macrophages could be crucial for the inhibition of OS progression, contrary to other solid tumors, and then useful in stratifying OS patients at diagnosis [20]. On these bases, targeting the immune cell context of TME could certainly represent an effective and very innovative therapeutic strategy for OS [16].

Nowadays the standard therapeutic strategy for OS patients is represented by neoadjuvant chemotherapy (pre-operative), surgical resection (limb amputation or most frequently limb-sparing surgery), and adjuvant chemotherapy (post-operative) [21]. The most used chemotherapy regimen for children is the combination of high-dose methotrexate, doxorubicin, and cisplatin (MAP) [9,22]. OS is characterized by high heterogeneity, chemo-resistance and complex interactions with the surrounding bone microenvironment [15,23,24], letting the available chemotherapeutic drugs not be completely decisive. Moreover, the necessary high dose of anticancer drugs often leads to many acute and chronic side effects [25], hence the identification of novel strategies to ameliorate the disease outcome and the patients’ life expectancy are necessary.

This review offers an overview on new possible innovative therapeutic strategies to better manage OS and, above all, ameliorate the outcome for patients overcoming both chemotherapy resistance and side effects that they frequently develop. In particular, we focused our attention on the alternative use of already known drugs, on the potential anticancer effects of some immune-modulators and also on the beneficial antineoplastic role of iron chelators also in OS, as observed for other cancers.

## 2. Therapy in OS

Prior to the 1970s, OS treatment was principally represented by surgical intervention (amputation or limb-sparing surgery), with 20% of 5-year survival rate [26]. Since then, therapeutic strategies also incorporated chemotherapy with specific drug combinations: MAP (high dose methotrexate, doxorubicin, and cisplatin), IE (ifosfamide and etoposide), bleomycin and vincristine, leading to an increase of survival rates to 67% for the patients with localized OS [8,9,10]. Standard treatment regimen for newly diagnosed OS currently includes neoadjuvant preoperative chemotherapy followed by surgical removal of the primary tumor and then postoperative adjuvant chemotherapy [9]. Multiple clinical trials were needed to define this management strategy. The Multi-Institutional Osteosarcoma Study (MIOS) is one of the most significative clinical studies in demonstrating the superiority of surgery plus chemotherapy compared with surgery alone [27]. It lasted from 1982 to 1984 and highlighted an 11% 6-year survival rate for patients who received only surgery versus a 61% 6-year survival rate for those who received surgery plus adjuvant chemotherapy [27]. In the same period at Memorial Sloan Kettering Cancer Center the neoadjuvant chemotherapy was introduced in the T10 protocol, in order to gain an extra time for the production of prosthetic devices and thus leading the increase of 5-years survival rate to 65% [28]. Moreover, the Pediatric Oncology Group compared neoadjuvant and adjuvant chemotherapies in a randomized study, demonstrating that they have a similar outcome [27]. Since that moment, preoperative chemotherapy became the standard with all its related advantages: extra time to plan surgery, easier tumor removal, and increased number of the good responders [13]. For patients with metastases, the 5-year survival rate is inversely proportional to the degree of metastatic disease and still remains at 20% [29]. They are currently treated with systemic neoadjuvant MAP chemotherapy, surgical removal of all visible lesions and adjuvant high-dose chemotherapy, but an extra effort is necessary to ameliorate their outcome. OS shows high heterogeneity, chemo-resistance, and complex interactions with the surrounding bone microenvironment [23,30,31], letting all the available chemotherapeutic drugs not be completely decisive. Moreover, the necessary high dose of anticancer drugs leads to many acute and chronic side effects [32]. Therefore, novel strategies are needed to improve both the therapeutic efficacy in OS and improve the life expectancy of all the patients.

## 3. Proteasome Inhibitors and Their Potential in OS

**Figure 1:**
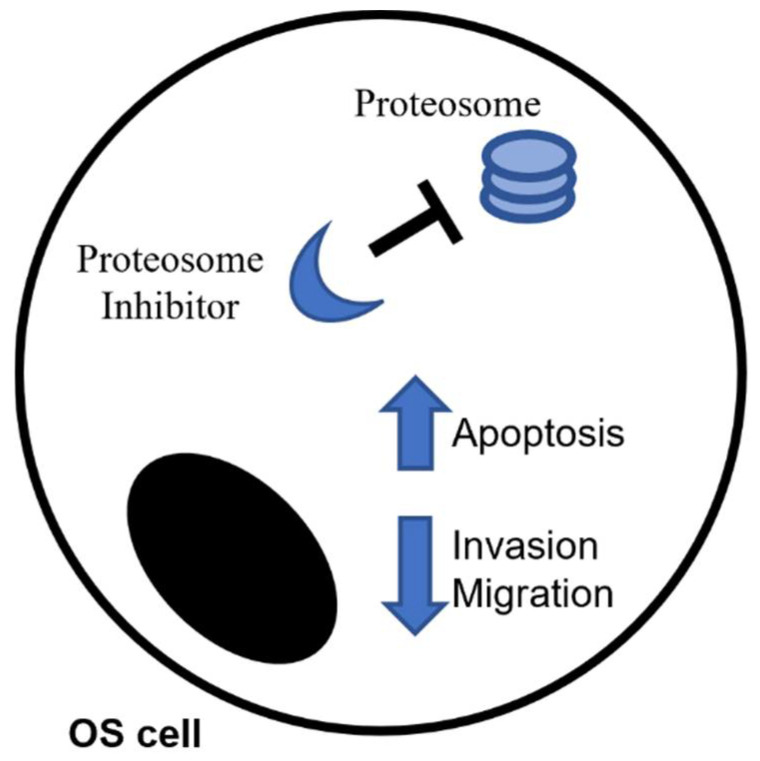
Proteasome inhibitors as anti-cancer drug in Osteosarcoma.

Proteasome inhibitors (PIs) are a class of compounds able to inhibit or limit the activity of proteasome, the multicatalytic complex normally responsible for proteins cleavage into peptides [33]. PIs are currently seen as very promising agents in oncotherapy due to the high sensitivity that tumor cells show towards them compared to healthy cells [34] (Figure 1). Their primary mechanism of action is certainly the inhibition of the proteasome, but the downstream events that then cause cell death are not completely clear. The induction of stress at the endoplasmic reticulum level and unfolded protein response (UPR) seem to be the principal underlying mechanisms [35], but a lot depends on the kind of cancer and on the specific type of agent. Several authors reported that PIs enhance apoptosis in multiple myeloma (MM) [36], indeed they are a standard part of front-line therapy in MM and mantle cell lymphoma, but also in leukemia [37] and some solid tumors, such as prostate tumor cells [38]. In 2017 Renhao Liu and collaborators, for example, demonstrated that MLN2238, a PI normally used in MM also in association with other drugs such dexamethasone [39,40], attenuates the invasive capabilities of two OS cell lines, by reducing the expression of MMP2/9 proteins [41]. In the same way other authors observed a significative accumulation of p53 after use of MLN2238 with the consequent activation of Caspases-3, -8, and -9-dependent cell death [41] in both OS [41] and other different tumors [42,43,44]. Bortezomib, the first PI approved by US Food and Drug Administration (FDA), also shows additional anti-tumor activity in vitro and in vivo in a few solid tumors, including OS [45,46]. Its anti-tumor activity is due to several mechanisms: the stabilization of the NF-κB inhibitor (IκB), the induction of misfolded proteins and the initiation of unfolded protein response (UPR) in the endoplasmic reticulum, which facilitates the clearance of misfolded proteins [47]. In addition to its antitumor function, Bortezomib also influences the bone remodeling by interfering with the RANK/RANK-L/OPG pathway, one of the most important system in the regulation of physiological bone homeostasis. In particular, it limits the RANK-L-induced OCs differentiation, through the modulation of p38, activator protein-1 (AP-1), and NF-κB pathways [48], and moreover seems to induce the osteoblasts (OBs) differentiation from human mesenchymal stromal cells (MSC) [49,50]. In literature there are evidences regarding both the involvement of RANK/RANK-L/OPG in the pathogenesis of several bone tumors (i.e., osteosarcoma, giant cell tumor of bone, chondroblastoma) and the possibility to inhibit RANK-L to reduce the tumor-induced lesions of bone often observed in cancer [51].

Unfortunately, the use of PI often causes resistance in patients [52], as well as thrombocytopenia, asthenia, nausea, and peripheral neuropathy, thus determining discontinuation or dose reduction [47]. To overcome these limitations several authors proposed to use these drugs in combination with cannabinoids. One of the first demonstration of the effectiveness of this combination was obtained in 2014 by Morelli et al., which observed that Bortezomib together with cannabidiol, a cannabinoid from Cannabis sativa with high affinity with CB receptors [53], induced cell death and arrested cell cycle progression in MM cell lines [54]. Some years later, Nabissi and collaborator also demonstrated a synergism between Δ9-tetrahydrocannabinol, the active element of Cannabis sativa, and the PI, carfilzomib, in increasing death and decreasing migration of MM cells [55]. In 2018, Punzo et al. confirmed the anticancer effects of Bortezomib in OS cell line, but also demonstrated for the first time a stronger effect when it was used in combination with JWH-133 and RTX, the agonists at CB2 receptor and at TRPV1 channel, respectively [56]. In literature, there are several evidences about the anticancer role of endocannabinoid/endovanilloid system, of which CB2 and TRPV1 are the main receptors, but only in 2017 was this role also observed in OS [57], attributed to the system capabilities able to modulate inflammatory processes that are very strongly connected with tumor progression [58,59,60].

## 4. Endocannabinoid/Endovanilloid System in OS

The Endocannabinoid/Endovanilloid (EC/EV) system is a neurotransmission system, responsible for intercellular communication and composed by the cannabinoid receptor type 1 (CB1), the cannabinoid receptor type 2 (CB2), a non-selective cation channel (TRPV1), their endogenous ligands and all the enzymes involved in biosynthesis, transport and metabolism of ECs [56,57,61]. CB1 is mainly expressed in the central nervous system, while CB2 is localized in the periphery principally on immune and bone cells [56,57,61].

**Figure 2:**
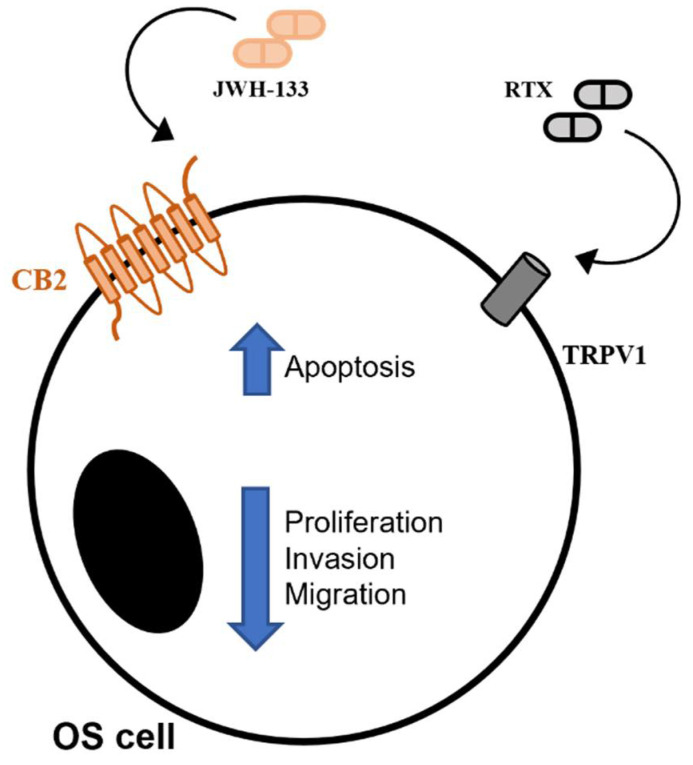
CB2 and TRPV1 stimulation induces anti-cancer effects in Osteosarcoma.

In literature there are several evidences about the anticancer role of EC/EV system [59,60], but only in 2017 was this role investigated for the first time also in OS [57] (Figure 2). In particular, it was demonstrated that the stimulation of both receptors CB2 and TRPV1 induced anti-proliferative, pro-apoptotic, and anti-invasive effect in six human OS cell lines, suggesting the EC/EV system as a new therapeutic target in OS [57]. The anticancer effects of EC/EV system are also related with its capabilities to modulate inflammation, the inflammatory status being connected with tumor progression [58,59,60]. Both CB2 and TRPV1 receptors show anti-inflammatory and anticancer properties when properly stimulated. CB2 receptor reduces inflammation [60], cell migration, and proliferation [60,61,62,63,64]. TRPV1 reduces the release of pro-inflammatory cytokines [65] and, moreover, the intracellular iron overload, related to its stimulation, is responsible for cell apoptosis by interfering with cell energy production and metabolism [66,67]. In literature, an important cross-talk between CB2 receptor and TRPV1 calcium channel is reported, observed principally in human osteoclasts (OCs) [68]. In particular, TRPV1 stimulation enhances the OCs activity (bone erosion), while CB2 stimulation promotes anti-osteoclastogenic events (bone deposition), suggesting these receptors as a new pharmacological target for conditions in which bone tissue is compromised (i.e., osteoporosis) [68,69,70]. On these bases it is possible to hypothesize a therapeutic anti-inflammatory and anti-tumoral role for the EC/EV system stimulation. Punzo et al. observed for the first time that several OS cell lines express EC/EV system elements and proposed the system as targets for OS therapy [57]. They stimulated CB2 receptor with its selective agonist, JWH-133, and TRPV1 with its vanilloid agonist, RTX, both safe in regard to possible psychotropic effects [57]. They observed an increase in apoptotic markers, a decrease in molecules responsible for proliferation and confirmed the capability to inhibit cell migration already reported in literature [55]. Considering these aspects, it is evident that EC/EV system stimulation could be useful to contain OS growth and expansion, but also, as an important secondary effect, to restore the equilibrium deposition/erosion of bone matrix, negatively altered from classical chemotherapy [64,70].

## 5. Immunotherapy and Its Potential in OS

**Figure 3:**
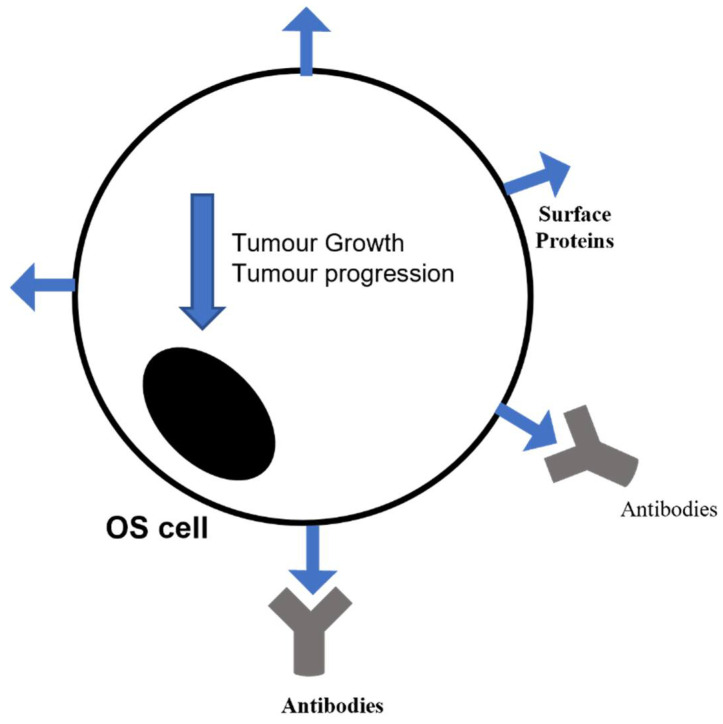
Immunotherapy as an innovative therapeutic approach to counteract cancer development and progression.

Immunotherapy is an innovative therapeutic approach used also in cancer with the purpose to help the immune system to counteract cancer development and progression (Figure 3). The immune system normally protects against foreign threats, such as infection or a tumor, and a tumor itself in particular limits the immune defense by releasing inhibitory cytokines or affecting the expression of protective markers [71]. In literature it is well documented the benefit derived from both innate immune cell-based therapy and immune stimulants associated with standard chemotherapy [72]. Additionally, OS is very susceptible to these therapeutic strategies [73], since it shows high levels of CD8+ infiltrating lymphocytes, positively correlated with the survival rate [20,74], and also for the presence on OS cell surface of markers targetable with selective antibodies [75].

Currently the most compelling evidence on immune modulation in OS regards the use of Mifamurtide (L-MTP-PE). In 2009 it was approved for the treatment of non-metastatic OS, in association with the standard therapeutic protocols [76,77], with an improvement in the 6-year survival rate from 70% related to the only chemotherapy to 78% [78]. L-MTP-PE activates macrophages and monocytes [77] thus exerting an immune system-mediated anticancer effect. In 2020 Punzo et al. observed a reduction in OS progression both by means of L-MTP-PE activated macrophages and, interestingly, by its direct effect on MG63 cells [79]. In particular, they demonstrated that L-MTP-PE not only exerts anti-neoplastic activity in OS, but also induced a M1/M2 intermediated macrophage phenotype switch, promoting a balance between pro-inflammatory and immunomodulatory macrophages functions [79]. L-MTP-PE is also able to influence bone metabolism, inducing an anti-osteoporotic effect in chemotherapy-induced osteoporosis in children with osteosarcoma [32]. According to this evidence, Punzo and collaborators observed that MG63 cells co-cultured with macrophages showed high levels of OPG and RANK-L, but when macrophages are activated with Mifamurtide they decrease, indicating an anti-osteoporotic effect of the drug [79]. It is known that poor prognosis in cancer is associated with a great vascularization of primary tumor mass and that tumor cells secrete angiogenic factors, among which Interleukin 17 (IL-17) derived from CD4+ T-cells; Mifamurtide reduces, both directly and through macrophage activation, the expression of IL-17R in MG63 OS cell line [79].

As regards the antibodies targeting cancer cell surface, they seem to also be very promising in OS therapy, for their safety and ready availability, but also for the effectiveness demonstrated in other pediatric cancers [80]. For example, olaratumab has been approved by Food and Drug Administration (FDA), as first-line treatment for soft tissue sarcoma in combination with doxorubicin [81], as well as the combination of carotuximab with pazopanib is under investigation in the TAPPAS phase 3 Trial on advanced angiosarcoma patients [82]. Following, the complex glembatumumab-vedotin is an antibody-drug conjugate directed against the osteoactivin, overexpressed on the OS cell surface [83], and cytotoxic to the OS cell lines [84] and also to other kinds of cancer, such as breast cancer [85,86]. Another antibody that is under investigations for OS is trastuzumab, initially developed for HER2+ breast cancer in combination with standard chemotherapy [87]. HER2 tyrosine kinase activity is essential in cell proliferation and differentiation, which is present also in OS cells, so it could represent a good anti-cancer target also in this solid tumor [88]. Targeting the surface proteins of OS cells is a very promising strategy and the trials investigating this possibility are in development, not only for the identification of good solutions, but also for drugs to avoid, such as denosumab. This is an antibody directed against RANK ligand, normally indicated for the pathologies characterized by bone compromission (i.e., osteoporosis, hypercalcemia, and osteogenesis imperfecta) [89]. Denosumab (DEN) finds application also in patients with bone metastasis from solid tumors, since its capability to reduce the risk of bone fractures [90]. Blay J. and collaborators in a Phase II study demonstrated that DEN totally (99%) inhibited the progression of giant bone cell tumor (GBCT) [91]. In the last years several studies have been performed to understand whether RANK-L/RANK/OPG pathway could have a key role also in OS. In 2015 Branstetter et al. observed the expression of RANK-L in human primary OS cells [92]. In blood samples derived from OS patients the RANK-L/OPG ratio is shifted in favor of RANK-L, with consequent weakening of a bone tissue already compromised by the tumor itself [93]. Another important consequence of this imbalance is the stimulation by RANK-L of RANK-positive OS cells, that seems to be related to cell migration. Indeed, Chen et al. observed that total invalidation of RANK-L arrested tumor development in an OS murine model [94]. Few years later, Navet and his research group inoculated RANK-expressing OS cells in several murine models, without observing any impact on cancer cell proliferation [95]. These studies suggest RANK-L and RANK as therapeutic trigger to manage OS, but on the other hand it can be hypothesized that this triggering would not directly affect the tumor. Bago-Horvath et al. examined 91 human OS and described RANK as a negative prognostic factor for survival. Indeed, the 70% of OS samples expressed RANK and its expression levels were associated with shorter survival and worse response to chemotherapy [96]. Even though many aspects must be clarified, the possibility of triggering RANK-L/RANK/OPG pathway to obtain therapeutic benefits against OS becomes more and more solid and concrete. Considering the involvement of RANK-L in tumor progression [97] and its presence on OS cell surface [98], in 2020 Punzo and collaborators tested DEN effects, alone and in combination with Doxorubicin, one of the most used anticancer drugs to treat OS, in OS cell line [99,100]. They discouraged the use of this antibody in OS, because it worsened the effect of standard chemotherapy [101]. Tumors use several pathways to resist immunotherapy, thus making the identification of the mechanisms underlying this resistance necessary as well as the most useful combinations of immunotherapy and conventional therapy.

## 6. Iron Chelation Effects in OS

Iron is a crucial element for mammalian cells responsible for several physiological processes, such as DNA and hemoglobin synthesis, cellular respiration, cell growth, and proliferation [102,103,104,105,106,107]. Iron metabolism is finely regulated by different proteins, such as transferrin (TF), transferrin receptor (TFR-1), ferritin, and ferroportin (FPN-1) [102,103,108]. In particular, TF is an iron-binding protein involved in circulating iron transport [109,110,111]. TF is recognized by its selective receptor, TFR-1, which internalized iron-TF compound [109,110,111]. Once inside the cell, iron could be stored by binding ferritin, or it could be released by cells through FPN-1, the only known iron exporter [111]. Defects in these compounds activity could induce iron accumulation in cells and consequently Reactive Oxygen Species (ROS) production [106,109,112,113,114,115]. ROS are involved in DNA, protein, and lipids damages (b) and also in tumorigenesis [106,109,112,116]. It is known that cancer cells request high concentration of iron for their survival and proliferation [109,117,118,119,120]. Indeed, iron excess is responsible for cancer onset, metastasis development and tumor microenvironment alteration [106,113,115]. Expression levels of several proteins involved in regulation of iron metabolism are strongly compromised in tumors, resulting in an increase iron influx [114]. In 2017 Green et al. observed that in renal cell carcinoma patients high TfR1 expression is correlated with chemoresistance [121], as well as its homologue TfR2 is up-regulated in several human cancer cell lines [122,123]. In cancer patients, intracellular FT level could be considered as prognostic factor, due to its increase and documented involvement in tumorigenesis [124,125]. In highly invasive tumors, FPN is lower and its levels are inversely correlated with patient survival [126]. Iron has an important role in cancer principally because it is able to modulate the immune response of macrophages on T-cells [127]. In the earliest stages of tumorigenesis, pro-inflammatory cytokines cause iron sequestration in macrophages, thus creating the first-line defense against neoplastic transformation. Anti-inflammatory macrophages M2 and lymphocytes simultaneously release iron within the TME [128]. Iron-release phenotype is associated with up-regulation of the iron exporter FPN, while the iron storage protein FT is downregulated [129]. As immediate consequence, the iron-donating macrophage phenotype enhances tumor cell proliferation and growth [130,131].

**Figure 4:**
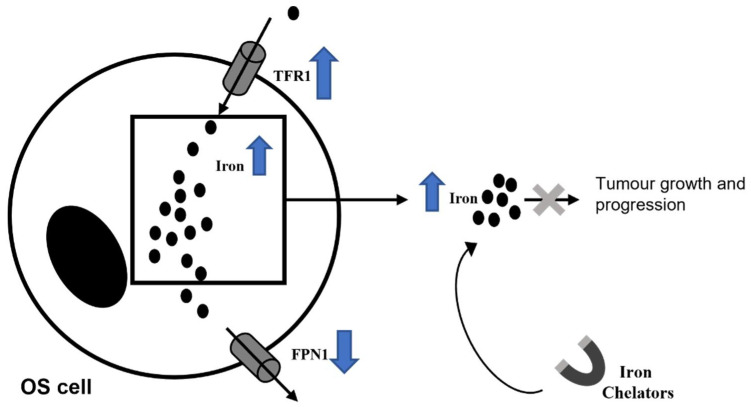
Iron chelators, by sequestering iron, exert anti-cancer properties.

Considering iron involvement in cancer progression, several authors proposed iron chelation as therapy to counteract tumor cell proliferation and growth, thus suggesting iron chelators as anti-cancer drugs [104,132,133,134] (Figure 4). For example, Deferasirox (DFX), the most common iron chelator used in iron overload diseases, shows anti-cancer activity by inhibiting the proliferation of cancer cells [132,133,134,135,136,137] emerging iron chelator [138,139], as an anti-neoplastic drug in hepatocellular cancer, and in acute myeloid leukemia [140,141,142]. Indeed, ELT mobilizes intracellular iron [139], thus inhibiting proliferation and inducing apoptosis of cancer cells [140,141,142,143]. Considering that an increase in intracellular iron concentration is observed in OS cells [104,112,144], several investigations aim to understand the role of iron chelators in the treatment of OS. Ni et al. have demonstrated that the iron chelator deferoxamine (DFO) was able to suppress tumor growth in a tumor-load model obtained by injecting SAOS-2 OS cell line in nude mice, confirming both roles of iron in promoting OS development and of iron chelator in counteracting tumor progression [104]. Furthermore, it has been demonstrated that the iron chelator di-2-pyridylketone-4, 4-dimethyl-3-thiosemicarbazone (Dp44mT) not only inhibits proliferation, migration, and invasion of 143 B OS cell line in vitro and of 143B xenograft in nude mice in vivo, but also exerts pro-apoptotic properties (Dp44mT) [144]. These data confirm once again iron involvement in OS progression and the beneficial effect of iron chelation. In contrast to these evidences it has been reported that in MG63 and 143B OS cell lines both the iron chelators DFX and ELT, alone or in combination, did not carry out anti-tumor activity through the modulation of the apoptotic and proliferative pathways in OS [112]. These conflicting data on the anti-cancer role of iron chelators in OS could be explained by considering their different mechanisms of action. Most likely ELT and DFX, unlike Dp44mT, could regulate other pathways or alter the tumor microenvironment, without affecting proliferation and apoptosis.

## 7. Emerging Anti-Neoplastic Drugs in OS

In recent years, several studies are underway to identify novel and more effective therapeutic strategies. In 2009 Psalia et al. described the implication of exosomes in the progression of several solid tumors and in the development of pre-metastatic niches, which can be colonized by cancer cells forming secondary tumors [145]. In addition, human OS cells have exosomes, expressing specific metalloprotease (MMP-1 and -13) involved in cell recruitment and cancer cell colonization, thus representing a good therapeutic target or also a good biomarker for prognosis [146]. In OS patients it has been observed a strong correlation between circulating angiogenic factors and poor prognosis [147], therefore the prevention of tumor angiogenesis represents an interesting and innovative therapeutic strategy. In 2017 Senerchia and collaborators published the results of a randomized, prospective clinical trial in which they tested the metronomic chemotherapy in high-grade non-metastatic OS [148]. Even though these results did not support the routine use of cyclophosphamide and methotrexate as metronomic agents, further investigation in metastatic diseases should not be precluded [148]. Moreover, in OS, over 25,000 aberrantly regulated long non-coding RNA (lncRNA) have been detected [149]. Among them H19, up-regulated in OS and involved in tumor development [150], HOTAIR and MALATl, both responsible for drug resistance, invasion, and metastatization [151,152]. These lncRNA could represent a valid therapeutic target. The research of novel therapies for OS focuses also on the possibility to use agents already known and used for other purposes. For example, the tyrosine kinase inhibitors (TKIs) are under investigations for a possible use in OS [153]. They are normally used in the treatment of cancers, such as hepatocarcinoma, because of their ability to inhibit the protein tyrosine kinases (PTKs). PTKs are molecules involved in signaling pathways of cellular differentiation and proliferation [154,155,156]. The most studied TKI is Sorafenib, which has been proposed by the Italian Sarcoma Group for the stabilization of the disease, and is able to block tumor growth, tumor angiogenesis, and tumor metastatic potential in preclinical murine models [157]. There is also Eribulin, a microtubule inhibitor that is associated with good response in OS xenografts and is also used in patients with breast cancer and soft tissue sarcoma [158]. In particular, in 2019 Kiyuna et al. observed that Eribulin was able to arrest tumor growth in an orthotopic xenograft model derived from a 16-year-old high-grade OS patient [158]. Glembatumumab vedotin and anti-disialoganglioside (anti-GD2) antibody have also been proposed as novel therapy in OS, since their capability to arrest tumor growth. Therapy anti-GD2 is normally used for neuroblastoma, and it is responsible for an improvement in event-free survival. The fact that almost all OS cells express GD2 is a promising indication to evaluate the possible efficacy of a therapy against GD2 also in this tumor [159].

## 8. Conclusions

Osteosarcoma is the most common malignant bone tumor, characterized by high metastasis rate and poor life quality [1,2,3,4]. The actual therapy for OS is represented by chemotherapy and surgical intervention, up to the amputation of the affected limb [1,160]. Despite the improvements that therapies can bring to survival, none are decisive and without side effects [9,22,26,161].

Therefore, the research of novel possible therapeutic strategies in OS is required not only to counteract its progression and its high grade of metastatic risk, but also the negative side effect related to standard chemotherapy. OS survivors are at risk of chronic health diseases (principally cardiovascular diseases and osteoporosis), chronic pain and physical limitation [162,163]. It is amply known that antineoplastic treatments affect metabolically active tissues such as bone and the cardiovascular system. The young adult OS survivors present bone mineral density that may predispose to osteoporosis (OP) of earlier onset than in normal population [32,164]. The cause of bone mineral density reduction has not been widely studied in OS survivors but there are clinical and experimental evidence of an association between increased pro-inflammatory cytokines activity and bone loss [165].

Exposure to chemo-and radiotherapy leads to inflammation and reactive oxygen species (ROS) accumulation. Inflammation stimulates the activation of cytotoxic mediators, such as ROS and reactive nitrogen species (RNS), which in turn stimulate the production of cytokines and adhesion molecules, and the activation and proliferation of lymphocytes generating a low-grade chronic inflammation responsible of osteoclast activation [166]. New therapeutic strategies are necessary to reduce OS long-term adverse effects such as OP and improve survivors’ health conditions. It has been reported that the EC/EV system stimulation has a key role in counteracting OS progression increasing apoptosis and decreasing both cell survival and invasion capacity in human OS [54]. In addition, the system is also involved in bone metabolism [58]. Osteoclasts from OS patients have reduced levels of the protective CB2 receptor compared with healthy subjects, and this condition is more marked when patients are undergoing chemotherapy [32]. The consequence is a decrease in bone mass density that could induce OP over the years. Therefore, it cannot be excluded a possible combination between CB2 and TRPV1 drugs and standard chemotherapy in OS to reduce cell survival and invasion capacity and to counteract the development of OP induced by chemotherapeutic agents.

As other tumors, OS shows a mutual dependence with tumor microenvironment (TME) [6,14,31], a system of surrounding cells, molecules, and factors involved in oncogenic transformation, angiogenesis, metastasis, survival, and resistance to chemotherapy [167]. TME is mainly composed by infiltrating immune cells, including tumor-associated macrophages (TAMs) [168]. TAMs are distinguished in classically activated M1, with pro-inflammatory and anti-tumor properties, and alternatively activated M2, with immune-suppressive and tumor enhancing properties [79]. In high-grade OS patients M2 polarized macrophages are responsible for the reduction of metastasis and for survival improvement, contrarily to other tumors in which they are associated with cancer progression [19]. Moreover, OS cells show a great iron (Fe) necessity to sustain their high metabolism rate, indeed they manifest an important internalization of Fe [104,112,144].

It is reported that proteasome inhibitors (PIs) enhance apoptosis in several tumors [169,170]. In particular, the PI MLN2238 reduces the invasion capability of two OS cell lines, reducing the expression of MMP2/9 proteins [41], and inducing cancer cells apoptosis, by activating Caspase-3, -8 and -9 in both OS [38] and other different tumors [42,43,44]. Bortezomib is used as anticancer drug in multiple myeloma and in lymphoproliferative neoplasms [46,171]. It exerts anti-cancer properties also in OS [45], by inducing apoptosis, reducing migration capacity, and arresting cell cycle progression in OS cell line [56]. Since it induces resistance and several adverse events in patients [47], it is proposed in combination with cannabinoids [56], known to have an anti-cancer activity in OS [57,58], in order to reduce its dose and side effects. BTZ showed a more marked effect when used in combination with JWH-133, selective agonists at CB2 receptor, and RTX, selective agonist at TRPV1 receptor [56]. It has been reported that the EC/EV System stimulation has a key role in counteracting OS progression, increasing apoptosis and decreasing both cell survival and invasion capacity in human OS [57].

Considering the protective role of the immune system in infections and cancer, immunotherapy seems to be a new therapeutic strategy to counteract tumor progression [71]. Mifamurtide, a synthetic analogue of bacterial liposome-encapsulated muramyl tripeptide phosphatidyl ethanolamine, is used for treatment of non-metastatic OS in combination with the standard therapy, improving the 6-year survival rate from 70% related to the only chemotherapy to 78% [29,76,78]. Recent studies highlighted the importance of Mifamurtide in preventing OS-associated osteoporotic events [32] and also in counteracting OS progression by intervening with the tumoral microenvironment modulating phenotype switch of tumor-associated macrophages [79]. It is able to induce a M1/M2 intermediate macrophage phenotype and it also exerts anti-invasive and anti-osteoporotic effects in OS [32,79].

The use of antibodies directed against proteins on tumor cells surface in combination with standard chemotherapy has proved to be a very promising strategy for inhibiting their survival and growth [80,81]. Glembatumumab-vedotin, an antibody-drug conjugate targeting the osteoactivin, overexpressed on the OS cell surface, seems to counteract cancer progression [83,84]. Trastuzumab could represent a good anti-cancer target in OS; it is an antibody directed against HER2 tyrosine kinase, which is essential in cell proliferation and differentiation [80]. Another antibody-drug being studied for the treatment of osteosarcoma is Denosumab, a fully human monoclonal antibody that binds RANK-L with high specificity and affinity and induces a reduction of OCs maturation and, consequently, of bone erosion [98,172]. RANK-L is involved not only in bone compromission, but also in cancer progression [89]. Unfortunately, Denosumab administered in combination with Doxorubicin worsened the effect of standard chemotherapy in OS cell lines, so its use is not recommended [99,100].

It is known that intracellular iron accumulation is responsible for cell damage, caused by Reactive Oxygen Species (ROS) production [97,100,103,104,105,106], which are involved in DNA, protein, and lipids damages and also in tumorigenesis [97,100,103,104,105,106,107]. Iron high levels induce cancer onset, metastasis development, and TME alteration [97,104,106]. Interestingly, considering the involvement of iron in tumor progression, iron chelators have been proposed to counteract cancer. There is conflicting evidence about the effects of iron chelators in OS [104,132,133,134]. It has been reported that the iron chelator Dp44mT inhibits proliferation, migration, and invasion of OS cell line [144]. Contrarily, it seems that the iron chelators DFX and ELT do not exert any anti-cancer activity in different OS cell lines [112].

However, other novel and more effective therapeutic strategies are currently under investigation to counteract OS progression, metastatic risk and also the side effects induced by chemotherapy. Currently, targeting OS aberrant long non-coding RNA (lncRNA), such as H19, HOTAIR, and MALATl, responsible for drug resistance, invasion and metastatization, is proposed to counteract OS progression [150,151,152,157]. However, tyrosine kinase inhibitors (TKIs) directed against protein tyrosine kinases (PTKs), which are involved in signaling pathways of cellular differentiation and proliferation, are under investigation in OS [153,155]. It has been reported that in preclinical murine models of sarcoma Sorafenib counteracts tumor progression, by inhibiting growth, angiogenesis, and metastatic potential [137]. Moreover, Eribulin, is associated with a good response in OS, inducing an arrest of tumor growth in a xenograft model [158].

In conclusion, in this review we described several novel possible therapeutic approaches for OS proposing several types of drugs with different molecular mechanism from proteasome inhibitors to EC/EV specific agonists, Immunomodulators, iron chelators, monoclonal antibodies and TKIs (Table 1). These strategies could ameliorate the outcome for OS patients overcoming both chemotherapy resistance and the side effects that frequently develop.

## Figures and Tables

**Table 1:** The table reported the Novel Therapeutic Strategies discussed.

Novel Therapeutic Strategies	
**Type of Drugs**	**Examples**
**Proteosome Inhibitors**	MLN2238, Bortezomib
**Endocannabinoid/Endovanilloid Receptors Agonists**	JWH-133, RTX
**Immune-Modulators**	Mifamurtide
**Iron Chelators**	Deferasirox, Deferoxamine, Dp44mT, Eltrombopag
**Monoclonal Antibodies**	Glembatumumab-vedotin, Trastuzumab
**Tyrosine Kinase Inhibitors (TKIs)**	Sorafenib, Eribulin

## Data Availability

Not applicable.
